# Psychosocial outcomes of folk dance interventions compared with other dance forms: a meta-analysis

**DOI:** 10.3389/fpsyg.2026.1930540

**Published:** 2026-09-23

**Authors:** Asuman Turan, Zeynel Turan

**Affiliations:** 1 Ph.D. Program, Institute of Educational Sciences, Burdur Mehmet Akif Ersoy University, Burdur, Türkiye; 2 Department of Turkish Folk Dances, Division of Turkish Folk Dances, State Conservatory of Turkish Music, Burdur Mehmet Akif Ersoy University, Burdur, Türkiye

**Keywords:** dance intervention, folk dance, meta-analysis, psychological well-being, psychosocial outcomes

## Abstract

**Introduction:**

This study aimed to determine the effects of folk dance interventions on individuals’ psychosocial outcomes and to compare these effects with those of other dance forms.

**Methods:**

A meta-analytic design was used, and experimental and quasi-experimental studies published between 2000 and 2026 were reviewed. A total of 20 independent studies met the inclusion criteria, of which 10 examined folk dance interventions and 10 examined other dance forms. Effect sizes were calculated using Hedges’ g, and the main analyses were conducted using a random-effects model. Subgroup differences were tested using a mixed-effects model, and the extent to which dance type accounted for between-study variance was examined through meta-regression.

**Results:**

The findings showed a positive, moderate, and statistically significant overall effect of dance interventions on psychosocial outcomes (*g* = 0.66, 95% CI [0.40, 0.92]). The pooled effect size was *g* = 0.58 for other dance forms and *g* = 0.76 for folk dance interventions. Although the effect size for folk dance interventions was numerically larger, the difference between the two subgroups was not statistically significant [Q_m_(1) = 1.06, *p* = 0.304]. Dance type accounted for approximately 37% of the observed between-study variance; however, the meta-regression coefficient was not statistically significant.

**Discussion:**

These results suggest that folk dance interventions and other dance forms may provide comparable benefits for psychosocial outcomes. Nevertheless, the findings should be interpreted cautiously because of the high level of heterogeneity, the limited number of studies, and the possibility of publication bias.

## 1 Introduction

Psychosocial health is a multidimensional construct that is not limited to the absence of symptoms of mental illness. It also involves individuals’ ability to derive satisfaction from life, regulate emotions effectively, establish healthy interpersonal relationships, and participate functionally in social life (Ryff, 1989, 2014; Keyes, 2002; Tsang et al., 2012). Accordingly, psychosocial health is commonly assessed through interrelated indicators such as depression, anxiety, stress, psychological well-being, quality of life, self-esteem, social skills, social functioning, and social cohesion (Keyes, 1998, 2005; Ryff and Singer, 2008; Lam et al., 2011; Brissos et al., 2011; Dykxhoorn et al., 2022; Topp et al., 2015). Previous studies have also shown that dance and folk dance practices may have positive effects on self-esteem, self-confidence, subjective well-being, socialization, and psychosocial development (Uslu, 2013; Saygılı and Özmutlu, 2019; Karabacak et al., 2021; Korkmaz and Çiftçi, 2021).

Physical activity has an important role in interventions designed to improve psychosocial health. Previous research has shown that regular physical activity can reduce depressive symptoms, lower anxiety, alleviate psychological distress, improve quality of life, and support overall well-being (Biddle and Asare, 2011; Schuch et al., 2018; Pearce et al., 2022; Singh et al., 2023; Noetel et al., 2024; Huang et al., 2024; World Health Organization, 2022). Some forms of physical activity are distinguished not only by their biological and physiological effects but also by psychosocial mechanisms such as social support, self-efficacy, social connectedness, and emotional interaction (White et al., 2024). Dance is one such practice (Hanna, 1987, 2008). By combining movement, rhythm, music, emotional expression, and social interaction, dance may support both physical and psychosocial development and can therefore be conceptualized not only as an artistic or recreational activity but also as a structured intervention for promoting psychosocial health (Burkhardt and Brennan, 2012; Koch et al., 2014; Sarıkaya et al., 2017; Tao et al., 2022; Sango and Pickard, 2024).

A substantial body of research has examined the psychosocial effects of dance interventions involving practices such as modern dance, creative dance, tango, salsa, zumba, and dance/movement therapy (Burkhardt and Brennan, 2012; Koch et al., 2014, 2019; Meekums et al., 2015; Sarıkaya et al., 2017; Karkou et al., 2019; Christopher et al., 2025). Systematic reviews and meta-analyses indicate that dance interventions may have positive effects on depression, anxiety, stress, quality of life, psychological well-being, and some cognitive and social-cognitive outcomes (Karkou et al., 2019; Koch et al., 2019; Podolski et al., 2023; Prudente et al., 2024; Fong Yan et al., 2024; Christopher et al., 2025; Jaldin et al., 2025; Zhang and Ma, 2025). Dance is therefore regarded as a multidimensional activity that integrates emotional expression, body awareness, interpersonal interaction, rhythmic coordination, and self-regulation processes (Hanna, 1987; Koch et al., 2019; Savidaki et al., 2020; Gomes et al., 2021).

In systematic reviews and meta-analyses of dance interventions, dance practices are usually treated as a broad intervention category. Although some studies distinguish between dance/movement therapy, recreational dance, social dance, traditional dance, or specific dance styles, limited attention has been given to folk dance interventions as a distinct category compared with other dance forms in relation to psychosocial outcomes (Koch et al., 2014, 2019; Meekums et al., 2015; Podolski et al., 2023; Fong Yan et al., 2024; Prudente et al., 2024; Jaldin et al., 2025; Zhang and Ma, 2025). Folk dance interventions differ from other dance forms not only in their movement repertoire but also in the cultural and social meanings they carry. Associated with cultural identity, belonging, community membership, and intergenerational transmission, folk dance practices mediate individuals’ interaction with both their physical and social environments (Dundes, 1984; Deagon, 2008; Karacan Doğan and Yetim, 2011; Smith, 2018; Piazzi and Harris, 2016; Price, 2019; Özbek, 2022; Shay, 2023). In this respect, folk dance interventions are not merely physical movement practices; they are also social practices through which cultural heritage, social memory, and local belonging are reproduced (Yıldırım et al., 2024; Śeker and Bolat, 2025; Yorgancıoğlu, 2025).

Collective movement, rhythmic synchrony, and synchronized behavioral patterns provide an important theoretical basis for explaining the psychosocial effects of folk dance interventions. Previous studies have shown that synchronized movement can strengthen social bonding, group intimacy, prosocial behavior, and group belonging (Tarr et al., 2015; Trainor and Cirelli, 2015; Launay et al., 2016; Tunçgenç and Cohen, 2016; Mogan et al., 2017; Fujiwara et al., 2020; Howard et al., 2021; Pizarro et al., 2022; Baranowski-Pinto et al., 2022; Suberry and Bodner, 2024). Features common to folk dances, such as adapting to a shared rhythm, performing common movement patterns as a group, and moving in physical coordination, may facilitate these mechanisms. Therefore, the psychosocial effects of folk dance interventions should not be understood solely as consequences of physical activity; they may also reflect interpersonal synchronization, coordinated group participation, and the social bonding processes generated through collective movement.

Although folk dance interventions share several psychosocial mechanisms with other dance forms, their emphasis on cultural identity, social belonging, and collective representation gives them distinctive characteristics. This underscores the importance of examining whether the psychosocial effects of folk dance interventions differ from those of other dance forms. A comparative analysis of folk dance interventions and other dance-based interventions may therefore address an important gap in the literature.

Accordingly, this study addressed the following research questions:

What is the overall effect size of dance interventions on individuals’ psychosocial outcomes?
What is the effect size of folk dance interventions on psychosocial outcomes?
What is the effect size of other dance forms on psychosocial outcomes?

Is there a statistically significant difference between the effect sizes of folk dance interventions and other dance forms on psychosocial outcomes?

## 2 Materials and methods

In this study, the multidimensional nature of psychosocial health was considered, and the relevant variables were evaluated within a common psychosocial outcome framework. Negative indicators such as depression, anxiety, and stress, together with positive indicators such as psychological well-being, quality of life, self-esteem, social connectedness, and social cohesion, were treated as outcome variables representing different but theoretically related dimensions of psychosocial health.

### 2.1 Study design

A meta-analytic design was used to examine the effects of folk dance interventions on psychosocial outcomes and to compare these effects with those of other dance forms. Meta-analysis is a research synthesis method that allows quantitative findings from comparable studies to be statistically combined and an overall effect size to be estimated (Borenstein et al., 2009; Card, 2012). The research process and reporting were guided by PRISMA 2020 (Page et al., 2021), and the study selection process is presented in Figure 1.

**FIGURE 1:**
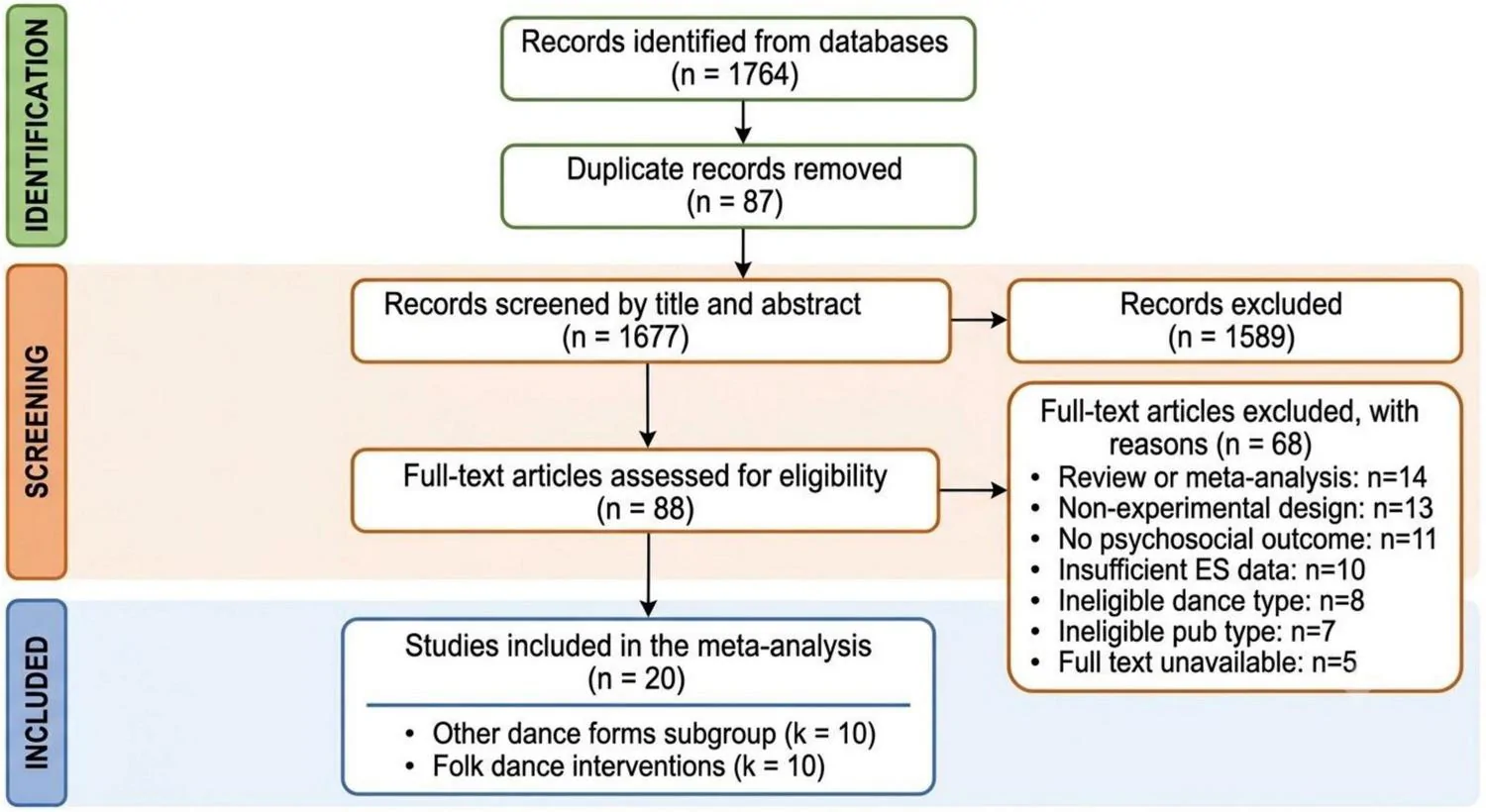
PRISMA 2020 flow diagram of the study selection process.

### 2.2 Databases and search strategy

A systematic literature search was conducted in Web of Science, Scopus, PubMed, ERIC, SPORTDiscus, ProQuest Dissertations & Theses Global, the YÖK National Thesis Center, and Google Scholar to identify studies relevant to the aim of this study. Studies published or completed in Turkish or English between 2000 and 2026 were considered. The searches were completed in May 2026.

The search strategy was developed around three conceptual blocks: dance type, intervention characteristics, and psychosocial outcomes. The terms “folk dance,” “traditional dance,” “ethnic dance,” “square dance,” and “circle dance” were used to identify folk dance interventions. Studies on other dance forms were identified using the terms “dance movement therapy,” “creative dance,” “modern dance,” “tango,” “salsa,” and “zumba.”

The terms “intervention,” “program,” “programme,” “training,” “exercise,” and “therapy” were used to capture the intervention component of the studies. For psychosocial outcomes, the search included “psychological well-being,” “psychological wellbeing,” “mental health,” “quality of life,” “self-esteem,” “depression,” “anxiety,” “stress,” “social connectedness,” and “social cohesion.” Searches were performed using the Boolean operators AND and OR.

The sample search string for folk dance studies was as follows:

(“folk dance” OR “traditional dance” OR “ethnic dance” OR “square dance” OR “circle dance”) AND (intervention OR program OR programme OR training OR exercise) AND (“psychological well-being” OR “psychological wellbeing” OR “mental health” OR “quality of life” OR “self-esteem” OR depression OR anxiety OR stress OR “social connectedness” OR “social cohesion”)

The sample search string for studies on other dance forms was as follows:

(“dance movement therapy” OR “creative dance” OR “modern dance” OR tango OR salsa OR zumba) AND (intervention OR program OR programme OR training OR therapy) AND (“psychological well-being” OR “psychological wellbeing” OR “mental health” OR “quality of life” OR “self-esteem” OR depression OR anxiety OR stress OR “social connectedness” OR “social cohesion”)

Searches were limited to titles, abstracts, and keywords according to the specifications of each database. Filters for publication year, language, and study type were applied using the available database options.

When developing the search strategy, relatively specific intervention-related keywords directly associated with psychosocial outcomes were preferred over broad dance-related terms. This approach was adopted to avoid unnecessarily expanding the results with publications not directly related to the research question, such as studies on dance education, performance, choreography, cultural representation, or theoretical dance research. Separate search blocks were used for folk dance interventions and other dance forms to preserve the comparative structure of the study.

### 2.3 Inclusion and exclusion criteria

Studies were included if they were published or completed between 2000 and 2026, examined an intervention based on folk dance or another dance form, evaluated at least one psychosocial variable such as psychological well-being, mental health, quality of life, self-esteem, depression, anxiety, stress, social connectedness, or social adjustment, and used an experimental or quasi-experimental design. Studies were also required to report sufficient statistical information for effect size calculation and to be available in full text. Reviews, systematic reviews, meta-analyses, book chapters, and conference papers were excluded. Postgraduate theses and experimental case studies were considered eligible if they provided quantitative data suitable for calculating effect sizes.

Folk dances were defined as practices based on the traditional dance repertoire of a specific society or cultural group. Practices such as modern dance, creative dance, tango, zumba, and dance/movement therapy were classified as other dance forms. Classification was based not only on the intervention’s name but also on its movement content, mode of implementation, and cultural context.

### 2.4 Data extraction and coding

The included studies were examined in detail. Author and publication year, country, research design, participant characteristics, experimental and control group sample sizes, dance type, intervention duration, session frequency, control condition, evaluated psychosocial outcome, and statistical data used to calculate the effect size were entered into a coding form. For studies classified as folk dance interventions, additional contextual information was extracted regarding the country or region in which the intervention was conducted and, where reported, the specific cultural, ethnic, national, or regional dance tradition represented by the intervention. These contextual characteristics were summarized to reflect the cultural heterogeneity within the folk dance intervention category.

When a study reported more than one psychosocial outcome, a single effect size was selected from each study to avoid the same sample being represented more than once in the analysis. A predetermined priority order was used to select the outcome variable. If a primary outcome was explicitly stated, that variable was selected. If no primary outcome was specified, the psychosocial variable most directly related to the study’s main aim was used. When more than one variable had the same priority level, preference was given to the outcome assessed with a standardized, validated measurement instrument.

Review or meta-analysis: *n* = 14

Non-experimental or quasi-experimental design: *n* = 13

No psychosocial outcome: *n* = 11

Insufficient effect size data: *n* = 10

Ineligible dance intervention: *n* = 8

Ineligible publication type: *n* = 7

Full text unavailable or duplicate sample: *n* = 5

### 2.5 Calculation of effect sizes

Results for continuous variables in the included studies were converted into a common metric using Hedges’ g. Hedges’ g was selected because several studies included relatively small samples.

The direction of effect sizes was coded so that positive values indicated psychosocial improvement in favor of the dance intervention. For variables in which higher scores indicated poorer outcomes, such as depression, anxiety, and stress, the direction of the effect was reversed. For variables in which higher scores indicated better outcomes, such as psychological well-being, quality of life, self-esteem, social connectedness, and social cohesion, the original direction was retained. Standard errors for effect sizes were calculated using the sample sizes and statistical information reported in the primary studies.

### 2.6 Quality assessment process

To evaluate the methodological characteristics of the primary studies included in the analysis, the researchers developed a structured methodological quality assessment form consisting of five criteria: K1, definition of participant selection and inclusion criteria; K2, random assignment to experimental and control groups or demonstration of baseline equivalence; K3, presence of a control group; K4, reporting of attrition or missing data; and K5, assessment of psychosocial outcomes using standardized measurement instruments. Studies meeting a criterion were assigned 1 point, whereas studies not meeting the criterion or providing insufficient information were assigned 0 points. Total quality scores ranged from 0 to 5.

Quality assessment was conducted independently by two researchers, and disagreements were resolved through consensus. This assessment was used to compare the methodological characteristics of the included studies and was not interpreted as a formal risk-of-bias tool. The results are presented in Table 1.

**TABLE 1:** Methodological quality assessment.

Study title	K1	K2	K3	K4	K5	Total score
Other dance forms						
Pinniger et al., 2012	1	1	1	1	1	5/5
Bahadır, 2022	1	0	1	1	1	4/5
Bräuninger, 2012	1	1	1	1	1	5/5
Rios Romenets 2015	1	1	1	0	1	4/5
Bracco et al., 2023	1	1	1	1	1	5/5
Koch et al., 2007	1	0	1	1	1	4/5
López-Rodríguez et al., 2017	1	1	1	1	1	5/5
Wang et al., 2020	1	1	1	1	1	5/5
Kaya Ciddi, 2021	1	0	1	1	1	4/5
Hackney et al., 2007	1	0	1	0	1	3/5
Folk dance ınterventions						
Jeon et al., 2005	1	1	1	1	1	5/5
Jeon and Choe, 2003	1	1	1	1	1	5/5
Mishra and Shukla, 2022	1	1	1	1	1	5/5
Serrano-Guzmán et al., 2016	1	1	1	0	1	4/5
Merom et al., 2016	1	1	1	1	1	5/5
Aydın and Akandere, 2017	1	0	1	1	1	4/5
Soykan and Mirzeoğlu, 2020	1	0	1	1	1	4/5
Kaltsatou et al., 2015	1	1	1	1	1	5/5
Solla et al., 2019	1	1	1	1	1	5/5
Tok and Güven, 2023	1	0	1	1	1	4/5

K1, selection criteria; K2, randomization/baseline equivalence; K3, control group; K4, attrition or missing-data reporting; K5, standardized measurement instrument.

The quality assessment results presented in Table 1 indicate that the methodological quality of the 20 independent studies included in the synthesis was generally acceptable. All studies had clearly defined selection criteria (K1), control-group designs (K3), and standardized psychosocial measurement instruments (K5), largely reflecting the review’s inclusion criteria. The main differences in methodological quality were related to the absence of randomization in some quasi-experimental and field-based studies (K2) and incomplete reporting of attrition or missing data in a small number of studies (K4). The concentration of scores between 3 and 5 suggests that the pooled effect size estimates were based on studies with relatively stable methodological features.

### 2.7 Data analysis

Meta-analytic calculations were conducted using JASP, an open-source statistical software package (Version 0.19.2). Standardized effect size estimates from the primary studies were converted to Hedges’ g, accounting for differences in sample size and potential bias in small-sample studies. The analysis matrix was weighted using the inverse-variance method. To maintain the assumption of sample independence, only one effect size from each study was included in the analysis. For studies reporting more than one psychosocial outcome, the priority order described in the section 2.4 “Data extraction and coding process” was followed.

Heterogeneity analyses were conducted to determine whether the observed variation across studies reflected sampling error or true differences in effects. Between-study variability was evaluated using Cochran’s Q statistic, Higgins and Thompson’s I^2^ index, and tau-squared (τ^2^), which estimates true effect variance. Because the included interventions differed in duration, session intensity, and participant characteristics, the effect sizes were assumed to follow a distribution of true effects rather than a single common effect. Therefore, the main analyses were conducted using a random-effects model. In addition, 95% prediction intervals were calculated to estimate the potential range of intervention effects in comparable future populations.

Subgroup analysis was conducted using a mixed-effects model to determine whether effectiveness differed by dance type (folk dance interventions vs. other dance forms). Because the subgroups appeared to have distinct heterogeneity structures and between-study variance was not homogeneous across layers, separate tau-squared estimates were calculated for each subgroup in JASP. The statistical significance of differences between pooled subgroup effects was tested using the between-group heterogeneity statistic (Q_m_) based on the chi-square distribution. In addition, a random-effects meta-regression analysis was conducted using Comprehensive Meta-Analysis (CMA) software to examine the explanatory contribution and directional trend of dance type as a categorical moderator. The explained variance ratio was reported as the R^2^ analog.

Publication bias was assessed to evaluate the robustness of the findings and potential systematic bias in the literature. Standard errors and observed effect sizes from the primary studies were visually inspected using a funnel plot. Potential funnel plot asymmetry was examined statistically using Egger’s linear regression asymmetry test and Begg and Mazumdar’s rank correlation test (Kendall’s tau). Rosenthal’s fail-safe N was calculated to assess the robustness of the pooled effect against unpublished or inaccessible studies with zero effects. All inferential statistical procedures were evaluated at α = 0.05.

## 3 Results

The methodological quality, sample characteristics, and statistical parameters of the primary studies included in the meta-analytic synthesis were organized in a systematic data matrix. Because Hedges’ g and standard error (SE) values had already been derived mathematically for the synthesized studies, this process did not involve any loss of data or effect size information. The final meta-analysis data matrix is presented in Table 2.

**TABLE 2:** Final data matrix used in the meta-analysis and subgroup analysis.

Group	References	Country/region	Dance form/tradition	Ne	Nc	Hedges’ g	SE
Other dance forms	Pinniger et al., 2012	Australia	Argentine tango	21	29	0.83	0.30
Other dance forms	Bahadır, 2022	Türkiye	Ballet	24	24	0.97	0.31
Other dance forms	Bräuninger, 2012	Germany	Dance/movement therapy	87	54	0.24	0.17
Other dance forms	Rios Romenets et al., 2015	Canada	Argentine tango	18	15	0.68	0.36
Other dance forms	Bracco et al., 2023	France	Tango therapy	13	13	−0.05	0.39
Other dance forms	Koch et al., 2007	Germany	Dance intervention	11	10	1.23	0.48
Other dance forms	López-Rodríguez et al., 2017	Spain	Dance-based intervention	42	53	0.56	0.21
Other dance forms	Wang et al., 2020	China	Chinese square dancing	33	33	1.08	0.26
Other dance forms	Kaya Ciddi, 2021	Türkiye	Recreational dance	12	12	−0.21	0.41
Other dance forms	Hackney et al., 2007	USA	Argentine tango	18	20	0.47	0.33
Folk dance ınterventions	Jeon et al., 2005	South Korea	Korean traditional dance	130	123	0.65	0.13
Folk dance ınterventions	Jeon and Choe, 2003	South Korea	Korean traditional dance	17	17	0.31	0.35
Folk dance ınterventions	Mishra and Shukla, 2022	India	Indian folk dance	20	20	−0.05	0.32
Folk dance ınterventions	Serrano-Guzmán et al., 2016	Spain	Dance therapy programme	35	32	0.58	0.25
Folk dance ınterventions	Merom et al., 2016	Australia	Social dancing	275	247	−0.09	0.09
Folk dance ınterventions	Aydın and Akandere, 2017	Türkiye	Folkloric dance	250	250	1.02	0.10
Folk dance ınterventions	Soykan and Mirzeoğlu, 2020	Türkiye	Folk dances	30	30	0.82	0.27
Folk dance ınterventions	Kaltsatou et al., 2015	Greece	Traditional dancing	16	15	1.38	0.40
Folk dance ınterventions	Solla et al., 2019	Italy (Sardinia)	Sardinian folk dance	10	9	1.80	0.54
Folk dance ınterventions	Tok and Güven, 2023	Türkiye	Folk dance instruction	20	20	1.89	0.38

Ne, experimental group sample size; Nc, control group sample size; Hedges’ g, standardized effect size coefficient; SE, standard error of the effect size.

Table 2 summarizes the data from the 20 independent studies included in the final analysis pool. The studies were organized to create a methodologically coherent data matrix, and coding was standardized in line with the analytical software’s requirements. To maintain the assumption of sample independence, one primary outcome-based effect size was extracted from each study. Ten studies were classified in the other dance forms subgroup, which included interventions such as modern dance, tango, zumba, and dance/movement therapy, whereas the remaining 10 studies represented the folk dance intervention subgroup, which included traditional and local dance practices.

The folk dance intervention subgroup represented culturally and geographically diverse practices rather than a single standardized dance tradition. As shown in Table 2, the included studies were conducted across different national and regional contexts and involved a range of traditional, folk, and socially embedded dance practices. These contextual differences should be considered when interpreting the pooled effect for the folk dance intervention subgroup.

The final matrix provided a data structure suitable for comparing the effect sizes of folk dance interventions and other dance forms through subgroup analysis and meta-regression. For the meta-regression models, the categorical group variable was dummy-coded numerically (folk dance interventions = 0; other dance forms = 1), with the variable name Group_Code. The significance level for all analytical procedures was set at α = 0.05.

The subgroup analysis results are presented in Table 3. Under the random-effects model, the other dance forms subgroup, which included modern dance, tango, zumba, salsa, and dance/movement therapy, showed a statistically significant and moderate pooled effect on psychosocial outcomes compared with control conditions (*g* = 0.58, 95% CI [0.27, 0.89], *t*(9) = 4.21, *p* = 0.002). Similarly, the folk dance intervention subgroup, comprising traditional and regional dance practices, showed a statistically significant moderate pooled effect on psychosocial outcomes compared with control conditions (*g* = 0.76, 95% CI [0.29, 1.24], *t*(9) = 3.62, *p* = 0.006).

**TABLE 3:** Effectiveness of folk dance interventions and other dance forms: subgroup effects and between-group comparison.

Analysis layer	Hedges’ g	95% CI	95% PI	t/Q_m_	df	*P*	I^2^ (%)	τ ^2^
Subgroup effects								
Other dance forms (*k* = 10)	0.58	[0.27, 0.89]	[−0.13, 1.29]	4.21	9	0.002	48.88	0.08
Folk dance interventions (*k* = 10)	0.76	[0.29, 1.24]	[−0.63, 2.16]	3.62	9	0.006	90.69	0.33
Difference between groups								
Folk dance interventions vs. other dance forms	–	–	–	1.06^b^	1	0.304	–	–
General model								
All studies (*k* = 20)	0.66	[0.40, 0.92]	[−0.30, 1.62]	5.33^a^	19	<0.001	80.75	0.20

k, number of independent studies; CI, confidence interval; PI, prediction interval; df, degrees of freedom; I^2^, percentage of true between-study variance; τ^2^, tau-squared variance. Within-subgroup heterogeneity was Q(9) = 18.19, *p* = 0.033 for other dance forms and Q(9) = 100.00, *p* < 0.001 for folk dance interventions. Superscript a indicates the t statistic for the overall model. Superscript b indicates Cochran’s Q_m_ statistic testing the difference between subgroups under the mixed-effects model.

Heterogeneity was present in both subgroups. The other dance forms subgroup showed moderate heterogeneity (*Q* = 18.19, *p* = 0.033, I^2^ = 48.88%, τ^2^ = 0.08), while the folk dance intervention subgroup showed high between-study variability (*Q* = 100.00, *p* < 0.001, I^2^ = 90.69%, τ^2^ = 0.33). These heterogeneity values suggest that the effects of dance interventions may vary across participant characteristics, session frequency, total intervention duration, and other methodological features of the primary studies. In addition, the 95% prediction intervals indicated that both dance forms covered a wide range of possible effects and that, at the population level, some future applications may yield effects near zero or in a negative direction (other dance forms PI [−0.13, 1.29]; folk dance interventions PI [−0.63, 2.16]).

Whether the effect sizes of folk dance interventions and other dance forms differed with respect to psychosocial outcomes was examined using the between-group heterogeneity test (Q_m_) within a mixed-effects model. The difference between traditional folk dance interventions and other modern or creative dance forms was not statistically significant [Q_m_(1) = 1.06, *p* = 0.304; Table 3].

The meta-regression analysis under the common-variance constraint showed a positive coefficient for the folk dance category; however, it did not reach statistical significance (β = 0.3842, *p* = 0.0772). Dance type accounted for approximately 37% of the observed between-study variance. Given the limited number of studies and the nonsignificant coefficient, this result should be interpreted as exploratory.

Funnel plot asymmetry and related statistical tests were conducted to assess the robustness of the overall effect size and the potential for systematic bias in the literature. The findings are presented in Table 4.

**TABLE 4:** Tests of publication bias and effect robustness.

Statistical test name	Value	*P*
Rosenthal’s fail-safe N	490	<0.001
Kendall’s tau	0.163	0.369
Egger’s linear regression asymmetry test	2.190	0.029

The funnel plot of standard errors and observed effect sizes from the 20 independent studies (Figure 2) showed some asymmetry, particularly in the lower-left and lower-right regions outside the graph’s central boundaries. This visual asymmetry was supported by a significant Egger’s linear regression test (Egger = 2.190, *p* = 0.029). A significant Egger test at α = 0.05 suggests the possible presence of publication bias or small-study effects in the analysis pool. In contrast, Begg and Mazumdar’s nonparametric rank correlation test did not indicate significant asymmetry (Kendall’s tau = 0.163, *p* = 0.369).

**FIGURE 2:**
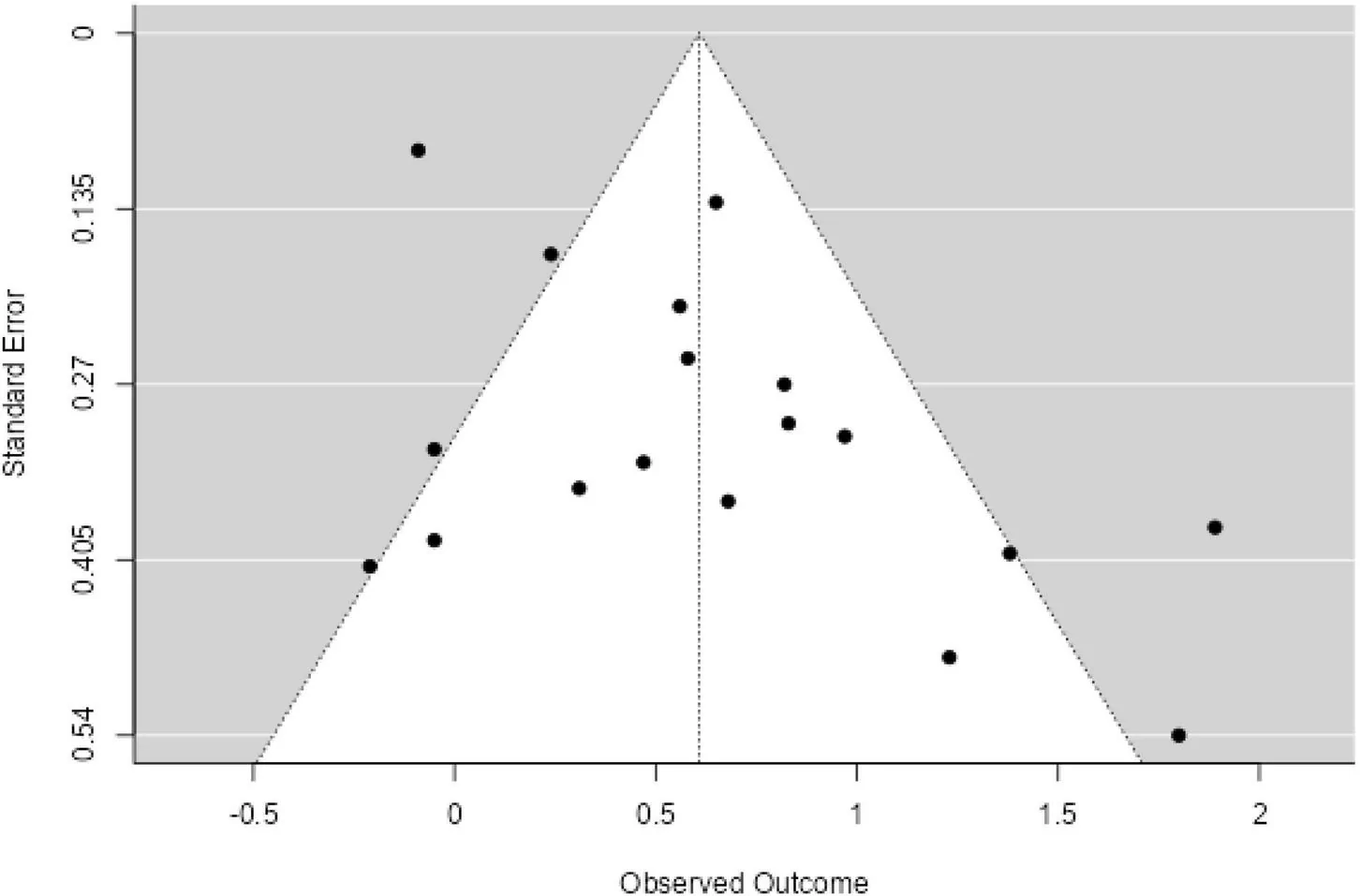
Funnel plot on the general effects of dance interventions on psychosocial outcomes.

Rosenthal’s fail-safe N was 490 (*p* < 0.001), exceeding the threshold of 110 calculated using the 5k + 10 criterion. This finding suggests some degree of statistical robustness for the overall effect. However, the significant Egger test (*p* = 0.029) and the nonsignificant Begg and Mazumdar test (*p* = 0.369) do not provide fully consistent evidence regarding publication bias. Therefore, the possibility of publication bias or small-study effects cannot be ruled out, and the Fail-safe N result should not be interpreted as conclusive evidence for the absence of publication bias.

The direction, relative weights, and pooled summary estimates of the effect sizes from the 20 independent studies were visualized in the forest plot presented in Figure 3. Each black square represents a primary study; the square’s size indicates the study’s weight in the analysis, and the horizontal lines represent the 95% confidence intervals (CIs). The pooled effect sizes for the subgroups and the overall model are displayed as diamonds on the horizontal axis. The midpoint of each diamond represents the pooled point estimate, and its left and right ends represent the 95% CI limits. The thick black horizontal lines below the diamonds indicate 95% prediction intervals (PIs), representing the potential range of intervention effects in comparable future populations.

**FIGURE 3:**
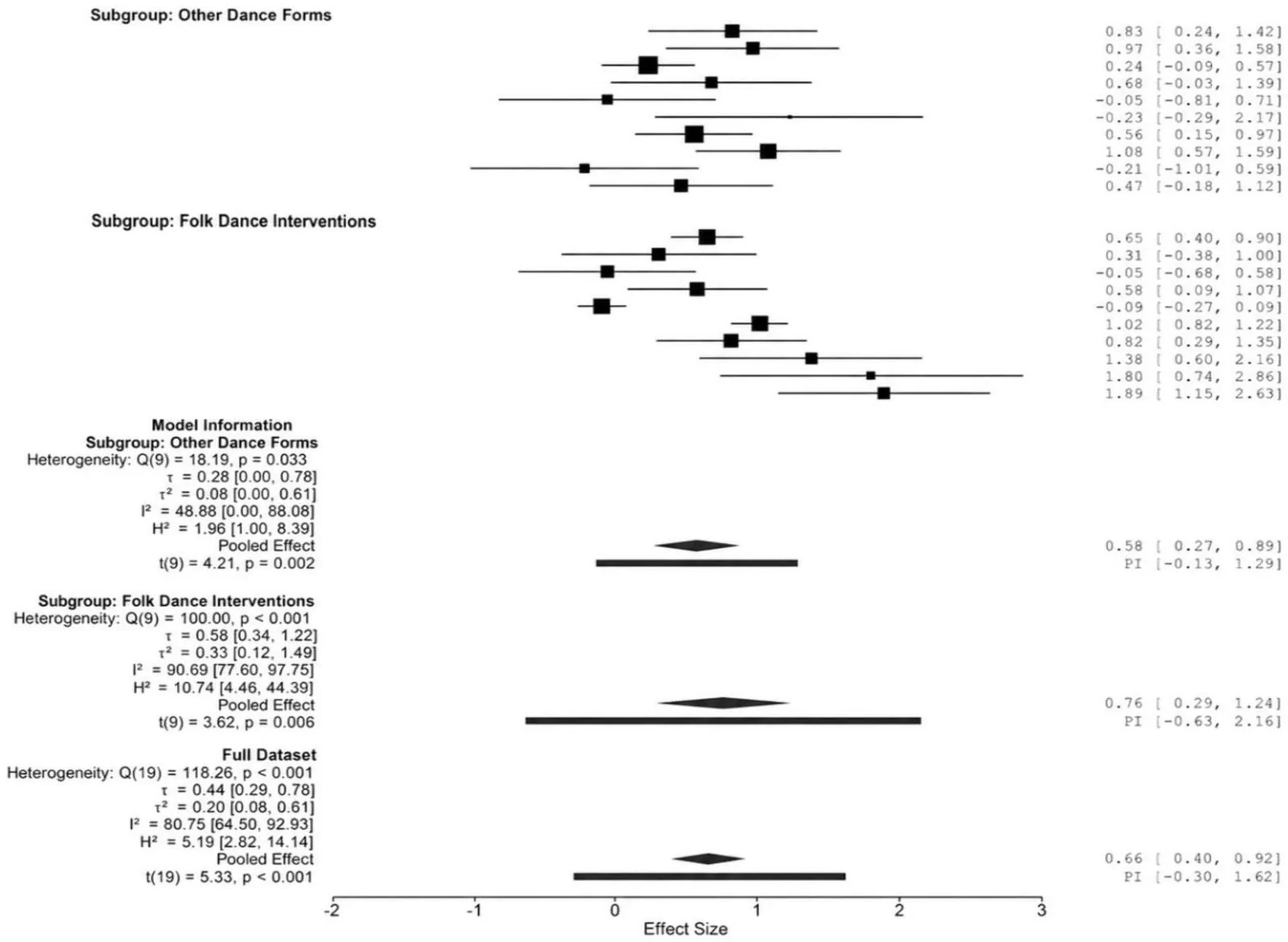
Subgroup and overall model forest plot of the effects of dance interventions on psychosocial outcomes.

In Figure 3, the 10 studies in the other dance forms subgroup are clustered within a relatively narrow range, and the pooled diamond indicates a moderate positive effect (*g* = 0.58, 95% CI [0.27, 0.89]). By contrast, the 10 studies in the folk dance intervention subgroup show wider dispersion along the horizontal axis (from *g* = −0.09 to *g* = 1.89). This broader distribution is reflected in the wider confidence interval for the pooled folk dance effect and the higher true between-study heterogeneity (*g* = 0.76, 95% CI [0.29, 1.24]). The confidence intervals for the two subgroup effects overlap substantially. Consistent with this visual pattern, the between-group heterogeneity test indicated that the subgroup difference was not statistically significant [Q_m_(1) = 1.06, *p* = 0.304].

The diamond at the bottom of the graph represents the overall model, which synthesizes all 20 studies in the analysis pool. Because the overall diamond is positioned entirely to the right of the vertical line of no effect, dance interventions showed a statistically significant positive mean effect on psychosocial outcomes compared with control conditions (*g* = 0.66, 95% CI [0.40, 0.92]). However, the prediction intervals for both subgroups and the overall dataset extend into negative values (overall dataset PI: [−0.30, 1.62]), suggesting that future studies may yield null or negative effects depending on the methodological design, participant characteristics, or intervention context.

## 4 Discussion

### 4.1 Overall effectiveness and theoretical foundations

This meta-analysis examined the overall effectiveness of folk dance interventions and other dance-based interventions on psychosocial outcomes compared with control conditions and evaluated whether these effects varied by intervention type. The findings showed that dance-based interventions produced a statistically significant moderate effect on psychosocial outcomes, including improvements in psychological well-being and reductions in depression, anxiety, and psychological stress. This finding is consistent with previous research indicating that dance interventions may have beneficial effects on psychological well-being, quality of life, depression, anxiety, and other psychosocial outcomes (Koch et al., 2019; Tao et al., 2022; Fong Yan et al., 2024; Prudente et al., 2024; Christopher et al., 2025).

These positive psychosocial effects may be explained by the combined physical, emotional, and social characteristics of dance. Previous studies have reported that regular physical activity can reduce depression, anxiety, and psychological distress, support mental health, and improve psychological well-being (Pearce et al., 2022; Singh et al., 2023; Huang et al., 2024; White et al., 2024). Dance also involves body awareness, emotional expression, and social interaction in addition to physical movement. Studies in dance and movement therapy suggest that dance can enhance body awareness, facilitate emotional expression, and support social relationships (Sarıkaya et al., 2017; Savidaki et al., 2020; Biondo, 2023; Tomaszewski et al., 2023). These mechanisms may help explain the positive psychosocial outcomes observed in the present meta-analysis.

### 4.2 Comparison of folk dance interventions with other dance forms: the role of collective synchronization

The subgroup analyses, one of the main methodological components of this study, showed that both the other dance forms subgroup, including modern dance, tango, zumba, and dance/movement therapy, and the folk dance intervention subgroup, including traditional and regional practices, produced significant improvements in psychosocial outcomes. The between-group comparison conducted under the mixed-effects model showed that the difference between the pooled effect sizes of the two intervention categories was not statistically significant (*p* = 0.304). This finding suggests that folk dance interventions and other dance forms may provide comparable benefits for psychosocial outcomes.

Although the meta-regression analysis under the common-variance constraint showed a larger effect size for the folk dance category than for the other dance forms category, this difference was not statistically significant (*p* = 0.0772). The relatively positive trend observed for folk dance interventions may be interpreted in light of collective synchronization and social belonging approaches in social psychology (Launay et al., 2016; Mogan et al., 2017; Fujiwara et al., 2020; Howard et al., 2021; Tarr et al., 2015). Folk dances may have distinctive psychosocial characteristics because they emphasize shared movement and group participation (Price, 2019; Liu, 2025; Karmas, 2023). Traditional folk dances often involve high levels of within-group interaction, simultaneous rhythmic coordination, and circular or linear formations performed hand in hand or arm in arm. Such interpersonal synchronization, supported by physical contact among participants, may strengthen group belonging and social bonding mechanisms (Launay et al., 2016; Mogan et al., 2017; Tunçgenç and Cohen, 2016; Suberry and Bodner, 2024). Performing culturally familiar rhythms and figures may also contribute to social cohesion and a sense of belonging by supporting collective self-esteem (Shay, 2023; Smith, 2018; Orel, 2025; Özbek, 2023; Yorgancıoğlu, 2025). These mechanisms may partly explain the relatively larger effect size observed in the folk dance intervention subgroup.

### 4.3 Heterogeneity structure and meta-regression implications

The high level of heterogeneity observed in the overall analysis pool, particularly in the folk dance intervention subgroup (I^2^ = 90.69%), is common in meta-analytic syntheses of behavioral and psychosocial interventions. The meta-regression analysis indicated that approximately 37% (R^2^ analog = 0.37) of the total between-study heterogeneity could be accounted for by adding dance type (folk dance interventions vs. other dance forms) to the model. This finding suggests that dance type may be one factor contributing to variation in effect sizes; however, this interpretation should remain cautious because the moderator coefficient was not statistically significant.

Moderate heterogeneity was observed in the other dance forms subgroup (I^2^ = 48.88%), and high heterogeneity was observed in the folk dance intervention subgroup (I^2^ = 90.69%). The distinct heterogeneity patterns across subgroups support the use of separate heterogeneity estimates in the analyses. The higher variance observed in folk dance studies may be related to differences in the movement characteristics of local dance traditions, the diversity of participant populations (e.g., children, adults, and older adults), and challenges in standardizing intervention content (Özbek, 2022, 2023; Orel, 2025).

### 4.4 Publication bias risks and small study effects assessment

Egger’s linear regression asymmetry test indicated statistically significant funnel plot asymmetry (*p* = 0.029). Although this may initially suggest potential publication bias, inspection of the data matrix suggests that the asymmetry may also reflect small-study effects. Several studies with relatively small sample sizes (Solla et al., 2019; Tok and Güven, 2023) reported large positive effect sizes, whereas larger, more methodologically controlled studies (Merom et al., 2016) reported near-zero or slightly negative effects. This pattern may have contributed to the significant Egger test result. The nonsignificant Begg and Mazumdar rank correlation test (*p* = 0.369), which is nonparametric and less sensitive to extreme values, provides some support for this interpretation.

Rosenthal’s fail-safe N suggested that the overall meta-analytic finding was relatively robust to the inclusion of unpublished null-effect studies. A Fail-safe N of 490 indicates that 490 additional independent studies with an effect size of zero would be required to render the overall positive effect statistically nonsignificant. Nevertheless, given the visual asymmetry in the funnel plot and the significant Egger test, these results should be interpreted cautiously. The observed asymmetry may not fully account for the overall findings, but the possibility of publication bias or small-study effects cannot be excluded.

## 5 Limitations and recommendations for future research

Despite its contributions, this meta-analysis has several methodological limitations that should be considered when interpreting the findings. First, the prediction intervals for the overall model and subgroups included zero, suggesting that dance interventions may not produce the same level of benefit in every future study. This indicates that factors such as intervention duration, participant characteristics, dance type, and control condition may influence the outcomes.

Second, although dance type accounted for 37% of the variance in the meta-regression model, 63% remained unexplained. Potential moderators, such as total intervention duration, weekly session frequency, session length, participants’ baseline clinical status (e.g., clinical depression or anxiety vs. healthy, sedentary populations), and age group, may contribute to this unexplained variance. Future meta-analyses should consider including these methodological and clinical variables simultaneously in multilevel meta-regression models and examining potential interaction effects.

Third, the meta-analysis included 20 studies in total, with 10 per subgroup. This limited number of studies is an important constraint, particularly for interpreting subgroup and meta-regression findings. The small evidence base may have reduced the statistical power to detect between-group differences and moderator effects. In addition, combining different psychosocial outcomes, such as depression, anxiety, psychological well-being, quality of life, self-esteem, and social adjustment, within the same analysis may have increased between-study heterogeneity. The combined inclusion of randomized controlled trials and quasi-experimental studies may also have contributed to methodological variability across studies.

## 6 Conclusion and recommendations

### 6.1 Conclusion

This meta-analysis synthesized data from 20 independent studies to determine the effects of folk dance interventions and other dance forms on psychosocial outcomes and to examine whether effectiveness varied by dance type. The random-effects model showed that dance interventions had a positive, moderate, and statistically significant effect on combined psychosocial outcomes compared with control conditions. This overall finding supports previous evidence indicating that dance-based physical activity may contribute to mental health and psychosocial well-being.

The subgroup analysis, which formed the central focus of this study, showed that both folk dance interventions and other dance forms had significant positive effects on psychosocial outcomes. Although the effect size for folk dance interventions was numerically larger, the difference between subgroups was not statistically significant. Therefore, the findings do not demonstrate that folk dance interventions are more effective than other dance forms; rather, they suggest that both categories may provide comparable psychosocial benefits. The meta-regression analysis showed a positive coefficient for the folk dance category, but this coefficient did not reach statistical significance (β = 0.3842, p = .0772). Although dance type accounted for approximately 37% of the observed between-study variance, this result should be interpreted as exploratory because of the limited number of studies. Finally, publication bias and robustness checks (Rosenthal fail-safe N = 490) suggested some degree of stability in the findings despite funnel plot asymmetry; however, the possibility of publication bias and small-study effects cannot be completely ruled out.

### 6.2 Recommendations

Based on the inferential statistics and methodological findings of this meta-analysis, the following recommendations are proposed for practice and future research:

Recommendations for practice

- Given the positive effects of traditional folk dance interventions on psychosocial outcomes and their potential to provide benefits comparable to those of other dance forms, folk dance interventions may be considered accessible and culturally acceptable psychosocial support practices in public education centers, local government programs, and recreational settings.
- Because folk dance interventions involve high levels of group interaction, rhythmic coordination, and collective synchronization, they may be considered complementary practices in psychosocial support programs designed for populations experiencing loneliness, social isolation, social-emotional adjustment difficulties, or mild to moderate depressive symptoms.
- Because the lower limits of the prediction intervals extended into negative values, dance interventions may not produce the same level of benefit in every application. Practitioners should therefore adapt intervention programs flexibly and sensitively according to participants’ age, physical capacity, and clinical status.

Recommendations for research

- Although dance type accounted for a substantial proportion of between-study variance in the meta-regression model, the remaining 63% of unexplained heterogeneity indicates the need for more standardized and transparent reporting of intervention dose (weekly session frequency, session duration, and total intervention weeks) and participant characteristics (clinical diagnosis, age group, and gender distribution) in future primary studies.
- To improve methodological quality and reduce the risk of asymmetry arising from small-study effects, future studies should use larger samples, active control groups, randomized controlled designs, and appropriate random assignment procedures.
- Future meta-analyses should, when sufficient data are available, conduct multilevel meta-regression and moderator analyses that include dance type together with relevant clinical and methodological variables. This approach may support the development of more specific, evidence-based intervention recommendations.

## Data Availability

The original contributions presented in this study are included in this article/supplementary material, further inquiries can be directed to the corresponding author.
